# Season, storage and extraction method impact on the phytochemical profile of *Terminalia ivorensis*

**DOI:** 10.1186/s12870-023-04144-8

**Published:** 2023-03-25

**Authors:** Aliu Moomin, Wendy R. Russell, Rachel M. Knott, Lorraine Scobbie, Kwesi Boadu Mensah, Paa Kofi Tawiah Adu-Gyamfi, Susan J. Duthie

**Affiliations:** 1 School of Pharmacy and Life Sciences, Robert Gordon University, Aberdeen, AB10 7GJ UK; 2 University of Aberdeen, Rowett Institute, Foresterhill Campus, Ashgrove Road West, Scotland, AB25 2ZD UK; 3 Department of Pharmacology, Faculty of Pharmacy and Pharmaceutical Sciences, Kwame Nkrumah University of Science and Technology, Kumasi, Ghana; 4 Faculty of Health and Allied Sciences, Department of Nursing and Midwifery, Pentecost University College, Accra, Ghana

**Keywords:** *Terminalia ivorensis*, Soxhlet extraction, Secondary metabolites, Phytochemicals, Season, Storage, LC MS/MS

## Abstract

**Background:**

*Terminalia ivorensis* (TI) is used in West African ethnomedicine for the treatment of conditions including ulcers, malaria and wounds. Despite its widespread use, the phytochemical profile of TI remains largely undetermined. This research investigated the effects of extraction method, season, and storage conditions on the phytochemical composition of TI to contribute towards understanding the potential benefits.

**Methods:**

TI bark was collected in September 2014, September 2018 and February 2018 during the rainy or dry seasons in Eastern Region, Ghana. Samples were extracted sequentially with organic solvents (petroleum ether, chloroform, ethyl acetate and ethanol) or using water (traditional). Metabolites were identified by liquid chromatography–mass spectrometry/mass spectrometry and compared statistically by ANOVA.

**Results:**

A total of 82 different phytochemicals were identified across all samples. A greater yield of the major phytochemicals (44%, p < 0.05) was obtained by water as compared with organic extraction. There was also a higher concentration of metabolites present in cold (63%, p < 0.05) compared with hot water extraction. A significantly (p < 0.05) higher number of phytochemicals were identified from TI collected in the dry (85%) compared to the rainy season (69%). TI bark stored for four years retained 84% of the major phytochemicals.

**Conclusion:**

This work provides important information on composition and how this is modified by growing conditions, storage and method of extraction informing progress on the development of TI as a prophylactic formulation or medicine.

**Supplementary Information:**

The online version contains supplementary material available at 10.1186/s12870-023-04144-8.

## Introduction

Plant secondary metabolites are of interest to the food and pharmaceutical industries due to their potential use in the prevention and treatment of various health disorders, as well as their use as dietary supplements [1]. Analysis of the molecular structures of secondary plant metabolites in plants can provide a potential mechanism for observed health benefits, as well as a route to isolation and/or synthesis at lower production cost than by isolating them from natural sources [2]. It also aids in the study of their efficiency, absorption, solubility, and stability in the human body [2, 3]. One notable example was the structural elucidation of salicylic acid which led to the synthesis of acetylsalicylic acid, the widely use non-steroidal anti-inflammatory [3].

*Terminalia ivorensis (TI*, Ivory Coast almond) of the family Combretaceae, is found in tropical and sub-tropical zones of the world [4, 5]. It is a large forest tree that grows up to 15–50 m in height and is branchless for up to 30 m [4]. TI trees are used commercially as a supply of solid timber for the building and construction industries and the wood for firewood and the production of charcoal [4–6]. Ghana’s economy benefited from exports of timber and timber products worth 73.35 million US dollars in 2021 and 134 million US dollars in 2020 [7]. The yellow extract from the bark is used as dye in the textile industry while the leaves have been reported to be a good material for producing conductive composites and an adsorbent for the sequestration of pollutants from the environment [4, 8].

Besides its economic usage, TI also serves as a good source of phytochemicals for ethno-medicinal purposes [9]. In in vitro studies, TI has been reported to show antibacterial, antifungal, antioxidant and anti-plasmodial effects [10–13]. Whereas several in vivo studies have found TI to possess anti-inflammatory, anti-nociceptive, anti-psychotic, hepatoprotective and nephroprotective properties in mice and rats [14–17]. In traditional medicine, TI is used in the West African region for the treatment of diuresis, general body pains, haemorrhoids, malaria, wounds and yellow fever [18–20]. However, there are limited data on the metabolite profile of TI and no information comparing the phytochemical profiles derived from traditional or more controlled chemical methods of extraction of TI. There are also no data showing the effect of herbalists’ preferential use of cold or hot extraction procedures as well as time of sampling on metabolites extracted from TI and hence the need to understand practice.

Practitioners of traditional medicine in Ghana usually employ the use of various solvents for extraction of plant products for therapeutic purposes including the proportional mixing of available solvents (such as alcohol, vinegar and water) soaking plant samples, such as flowers, leaves, bark or roots in these solutions at ambient temperatures overnight or boiling in water for several hours.

The climate of Ghana is tropical and characterised by rainy and dry seasons [21]. The northern part of the country has only one rainy season which occurs from April to September while the southern part of Ghana, where the study samples were obtained, has two rainy seasons which occur from April to July and from September to November. The national mean annual rainfall in Ghana is 1100–1900 mm. The dry season spans November to April [21–23]. There is approximately 12 h of daylight daily, and the temperature in the country fluctuates with season, with mean temperatures generally between 21 ºC and 35 ºC [24].

It is well established that seasonality impacts on the life cycle, distribution and composition of phytochemical in plants [25]. Changes in season, which are characterised by changes in light intensity, temperature, rain and wind patterns, affect plant morphology, flowering, fruiting, phytochemical profile and ability to compete with other species for survival [25, 26]. Being relatively immobile organisms, plants have developed alternative defence mechanisms to overcome stress conditions resulting from changes in weather, herbivory and microbial attack [26]. The production of a wide variety of secondary metabolites, including anthocyanins, cinnamic acids and flavonoids, is a major adaptation used by plants to overcome stressful conditions [25, 26]. The synthesis of secondary metabolites is closely regulated and restricted to specific plant tissues and developmental stages and is produced in response to stimuli such as reduced water, high temperature or decreased light intensity [27, 28]. Several studies have reported changes in secondary plant metabolites at the genetic or protein level due to stressful conditions [29–31]. For instance, decreased irrigation increased red beet total phenolics by 82% and by 98% in lettuce when copared with dequate water provision [32, 33]. Soil pollution with heavy metals such as cadmium, chromium and lead, have been shown to increase total phenolics (by 18 and 6%) and flavonoid content (by 12 and 7%) in *Ficus carica* and *Shinus molle* resectively when compared with samples from less polluted soils [34]. *Aloe vera* collected across different locations of India at varying altitudes, temperatures and rainfall patterns contain different amounts of alkaloids, flavonoids, glycosides, phenolic compounds, reducing sugars, saponins, steroids, tannins and terpenoids [25].

Herbalists usually store medicinal plant samples from days to several months in the chain of production of their medicines, particularly herbal bitters [35]. However, many plant metabolites are unstable and easily degraded or metabolised during storage [36]. For instance, flavonones are modified into anthocyanins in raspberry [37]. Moreover, phytochemical metabolites, such as α-tocopherol, benzoic acid, catechin, cyclohexen-1-carboxylic acid, lycopene, myoinositol and stigmasterol can be depleted during storage *in Cosmos caudatus* stored at room temperature for more than 12 h [36]. Investigating the impact of storage on metabolites within plant samples will provide important information for herbalists for designing sustainable plant harvesting, processing and storage techniques for medicine production [35] and will provide evidence-based knowledge on the “shelf-life” of plants used in traditional medicine [38].

This research investigated the comprehensive metabolite profile of TI and compared the aqueous method of extraction commonly used by traditional medical practitioners in Ghana with typical organic solvent extraction (i.e., sequential Soxhlet extraction). The research also compared the two commonly used traditional extraction methods: hot and cold-water extraction. This study further investigated the effects of season and storage on the phytochemical profile of TI.

## METHODS

### Collection and preparation of plant material

Fresh TI bark samples were collected from a forest in Asakraka Kwahu in the Eastern region of Ghana in both the dry (February) and wet (September) seasons of 2018. An additional sample from September 2014 which was stored for 4-years was also included to investigate the effect of long-term storage. Appropriate permissions were obtained for the collection of the plant and its use was executed in accordance with relevant guidelines. The samples were collected from trunks of TI trees assessed by a certified herbalist to be of approximately the same size and age and collected in an ethical and sustainable manner. The samples were authenticated by Dr George Henry Sam at the Department of Pharmacognosy, Faculty of Pharmacy and Pharmaceutical Sciences, Kwame Nkrumah University of Science and Technology (KNUST), Kumasi, Ghana and assigned a voucher herbarium specimen number KNUST/HEB/TI/SB/10/12 which was prepared and deposited in the department’s herbarium. The initial preparation of the samples was carried out at the Department of Pharmacology, KNUST and then transported at room temperature to the United Kingdom (Robert Gordon University and Rowett Institute) for further analysis. Immediately after harvesting the fresh TI bark samples, they were initially processed by washing them thoroughly with tap water and air drying them at room temperature for 2 weeks. They were then sent to the Rowett Institute for further processing and analysis. The dried samples were broken down into smaller pieces with a domestic food processor and then fine-powdered using a freezer mill (SPEX sample prep 6870, Fisher Scientific, Loughborough, UK). The fine powdered samples were vacuum sealed to minimise evaporation, oxidation and microbial growth and stored at -80 °C until required for extraction of phytochemicals.

### Extraction and identification of phytochemicals from TI samples

#### Hot water extraction of phytochemicals from TI

To mimic the traditional hot water extraction, TI sample (25 g, n = 3) collected in February 2018 was weighed into a conical flask and 250 mL of distilled water at ambient temperature was added with gentle swirling of the flask for 2 min to ensure that the sample was soaked properly without forming bubbles. The sample was heated on a hot plate (FB 15001, Fisher Scientific, UK) at 100 °C for 1 h. The extracted sample in the water solution was allowed to cool to room temperature and then filtered under vacuum through a 70 mm filter paper (FB 59017, Fisher Scientific, UK). The filtrate was frozen at -20 °C and freeze-dried (freeze-drier: Virtis Advantage EL, Biopharma Process Systems, Hampshire, UK) over 2 days to obtain dried powdered TI hot water extract for further analysis. After the completion of the hot water extraction, the residual TI was discarded and a new sample from the same batch of TI was used to repeat the extraction process. The samples were extracted in triplicates. These extracts were stored at -20 °C until required for analysis.

#### Cold water extraction of phytochemicals from TI

TI sample collected in February 2018 was prepared and soaked in distilled water as described earlier (Sect. 2.1) and extracted similarly to the traditional cold-water extraction. The conical flask containing the sample was covered with aluminium foil and allowed to stand for 48 h and extraction was carried out at ambient temperature. The content of the flask was filtered, freeze-dried, and stored as described earlier (Sect. 2.2.1).

#### Extraction of phytochemicals from TI samples using organic solvents

Sequential Soxhlet extraction is a common scientific method to extract and characterise secondary plant metabolites. Using automated Soxtherm equipment (Gerhardt Soxtherm, SX PC 1.40, Gerhardt, Germany) the procedure was performed for all three TI samples as described previously with some modifications [39]. Solvents were used in order of increasing polarity: petroleum ether (40–60 °C, extra dry, Fischer Scientific), chloroform (99.8+%, stabilized with ethanol, Fischer Scientific), ethyl acetate (99.98%, HPLC grade, Fischer Scientific), and ethanol (99.8%, absolute, Fischer Scientific). The freeze-dried TI sample (6 g) was weighed into each of the six cellulose thimbles and extracted with 140 mL of petroleum ether for 118 min at 150 °C. The remaining solvent was allowed to cool to room temperature and each of the extracts transferred into pre-weighed 25 mL round bottom flasks. Each replicate was evaporated under vacuum at 40 °C using a Buchi rotavapor (R-200, Sigma-Aldrich, UK) to obtain the petroleum ether extract of TI. The dried extracts were weighed separately to record yield before being pooled into a pre-weighed bottle, wrapped with tin foil to prevent light degradation and stored at -80 °C for further analysis. The TI residue left in the thimbles after the petroleum ether extraction was vacuum dried for 18 h at room temperature using a Heraeus vacutherm (VT 6025, Kendro, Germany) and reweighed. The drying was carried out in a vacuum to minimise oxidation. Using the specific extraction program on the Soxtherm machine for each solvent, the residue was sequentially extracted with chloroform (125 min extraction) with rotary evaporation at 62 °C, ethyl acetate (110 min extraction) with rotary evaporation at 77 °C and ethanol (115 min extraction) with rotary evaporation at 78 °C. After the completion of the sequential extraction, the residual TI was discarded and a new sample from the same batch of TI was used to repeat the extraction process. All samples were extracted in triplicates.

### Identification of phytochemicals isolated from TI samples

The method used for the identification of phytochemicals was as described previously [40]. Extracts from samples (10 mg/mL) were prepared in methanol (with 0.1% cetic acid) and 20 µL of each suspension was added to 40 µL of an internal standard and 40 µL of methanol. The internal positive standard was 2-amino-3,4,7,8-tetramethylimidazol [4,5-f] quinoxaline (100 ng/µL) and the negative standard was ^13^ C benzoic acid (400 ng/µL). The samples were centrifuged for 3 min at 10,000 x g at 4 °C, and the supernatant was subjected to liquid chromatography mass spectroscopy / mass spectroscopy (LC MS/MS) analysis. Phytochemicals were separated by liquid chromatography using an Agilent 1100 HPLC system with Zorbax Eclipse 5-µm, 150 × 4 mm column (Agilent Technologies, Wokingham, UK).

Three-gradient elution method was used with the mobile phase solvents as water and acetonitrile containing 0.1% acetic acid. An injection volume of 5 µL was used with a flow rate of 300 µL/min. The liquid chromatography eluent was directed into an ABI 3200 triple quadruple mass spectrometer (Applied Biosystems, Warrington, UK) fitted with a turbo ion-spray source. The mass spectrometer was run in a negative-ion mode for the analysis of indoles and phenolics with the settings: ion-spray voltage of -4500, source temperature of 400 °C, gases 1, 2 and curtain gas were set at 15, 40 and 10 respectively. For the analysis of heterocyclic amines, the mass spectrometer was run in positive ion mode with the settings: ion-spray voltage of 5500 V, source temperature of 400 °C, gases 1, 2 and curtain gas were set at 14, 40 and 10 respectively. All metabolites were quantified by multiple-reaction monitoring and ion transition for each of the analytes determined based on their molecular ion and a strong fragment ion. Differing elution times were used to overcome similarities in molecular ions and fragment ions for the different categories of compounds. Declustering potential, voltage variables, collision energy, collision cell entrance potential and collision cell exit potential were individually optimized for each analyte and the molecular weight were quantified in relation to the internal standards [40].

### Data analysis

**Fig. 1:**
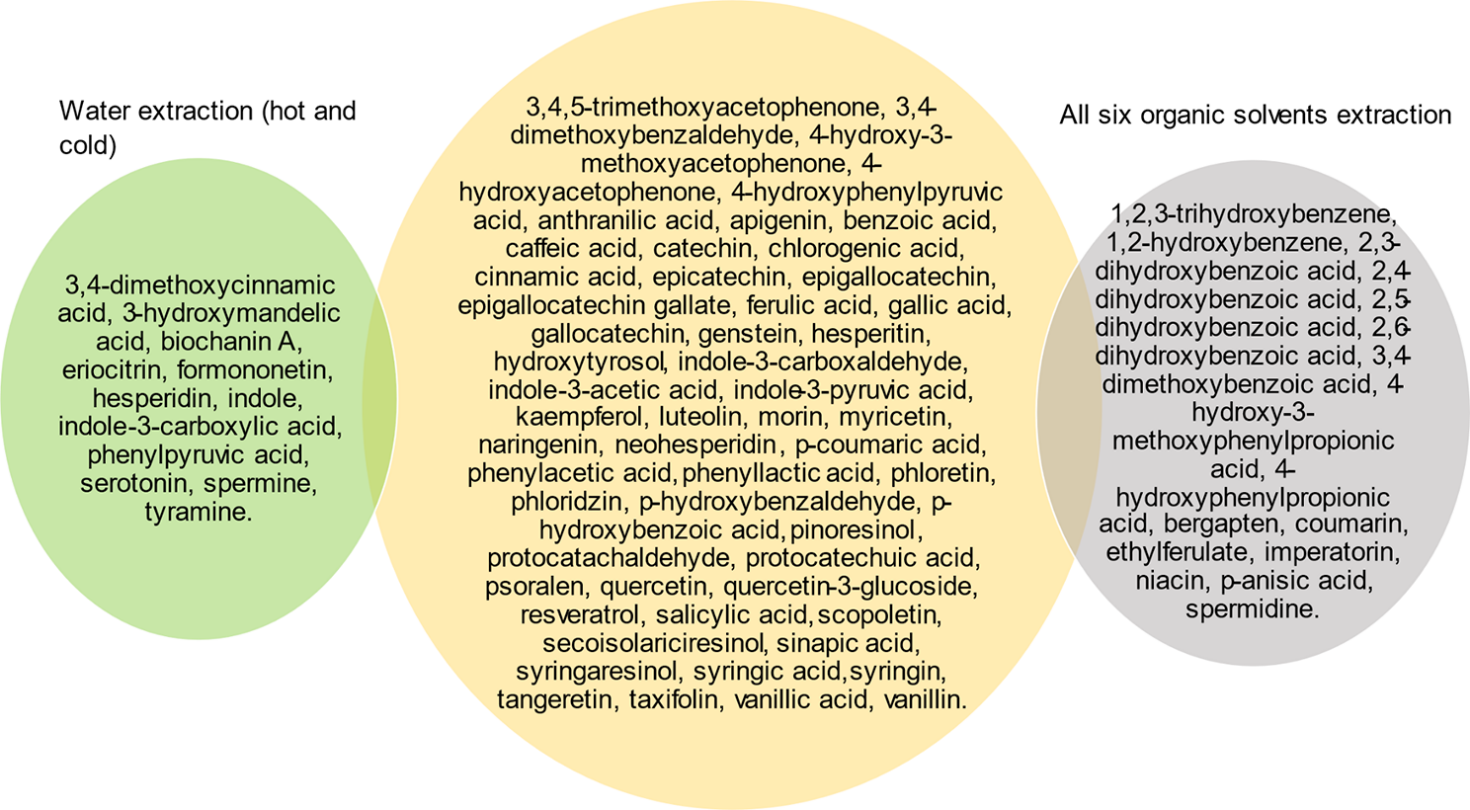
Distribution of phytochemicals between aqueous (combined hot and cold) extraction shown in green and all six organic solvents extraction shown in grey from the TI sample collected in the dry season (February 2018). Metabolites common to both extraction procedures is shown in gold

**Fig. 2:**
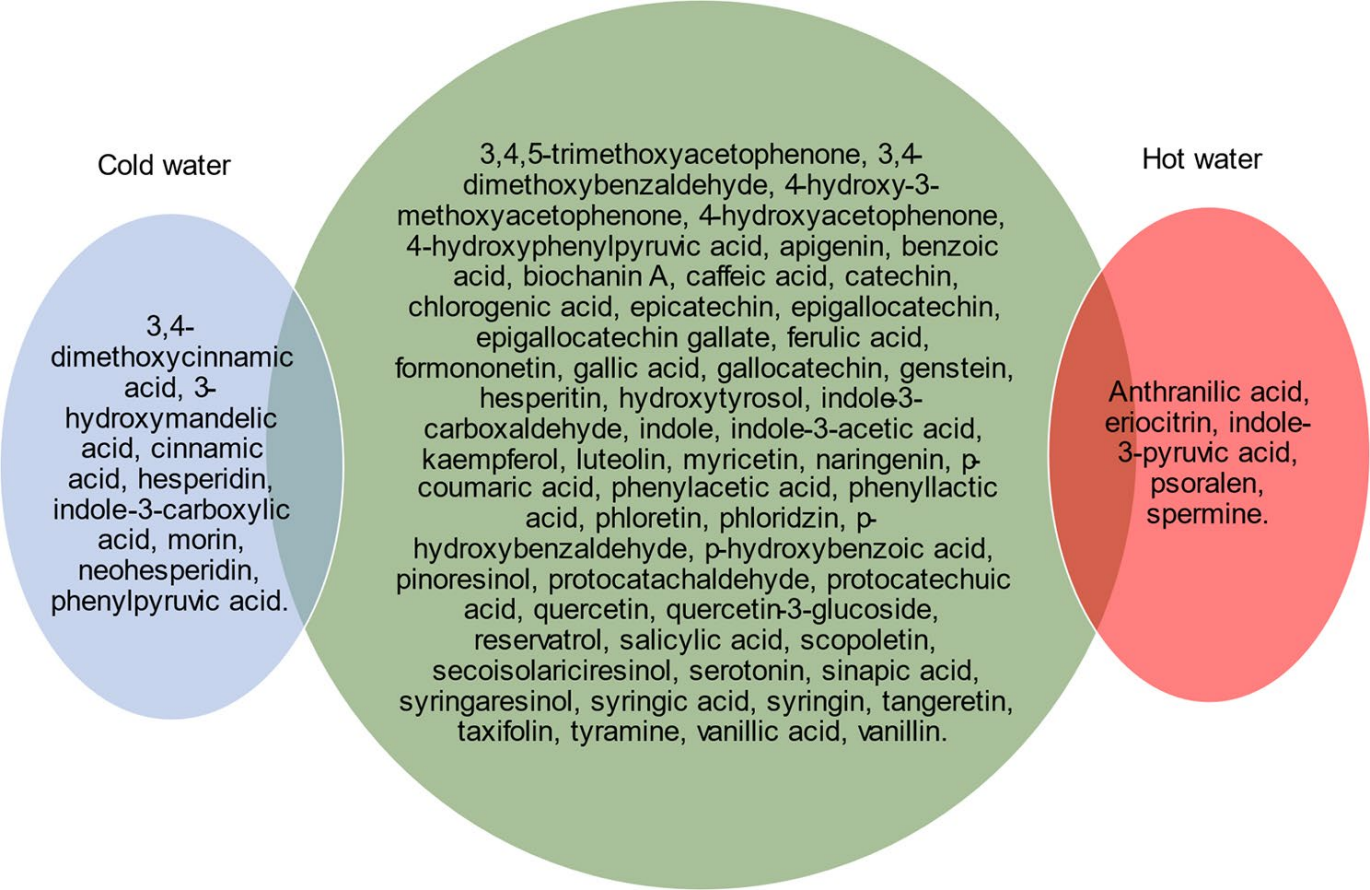
Distribution of phytochemicals between cold (blue), metabolites found in both extracts (green) or hot (pink) water extraction from TI sample (February 2018)

The identity and quantity of phytochemicals from TI (freshly obtained in February 2018) by organic solvent extraction was compared with phytochemicals recovered from aqueous extraction with the two commonly used traditional methods: hot and cold-water. Venn diagrams are used to visualise the relationship between the metabolites from the different extraction methods and to show the common phytochemicals that were compared by further statistical analysis (Figs. 1 and 2). Comparisons were made between the samples collected in September 2018 (rainy season) versus February 2018 (dry season) to assess the impact of season on phytochemical profile, and in September 2014 versus September 2018 to assess the impact of storage on phytochemical profile. These comparisons were made for the organic solvent extraction methods using petroleum ether, chloroform, ethyl acetate or ethanol. The concentration of phytochemicals present in the different samples were compared using analysis of variance (ANOVA) followed by Bonferroni’s post hoc test, with p < 0.05 considered as significant. TI samples obtained in September 2014, February 2018 and September 2018 were represented as 1, 2 and 3 respectively and a principal component analysis was carried out to determine the differences between the samples when extracted with the different solvents.

## RESULTS

### Effect of extraction solvent on the phytochemical profile of TI

#### Effect of organic versus water extraction on the phytochemical profile of TI

There was a total of 82 phytochemicals identified using both organic (chloroform, ethanol, ethyl acetate and petroleum ether) and water (cold and hot) extraction of TI obtained during the dry season. There was a greater number of metabolites isolated by organic solvent extraction as compared to water extraction. Phytochemicals including some amines (serotonin, spermine and tyramine), cinnamic acids (3,4-dimethoxycinnamic acid), flavonoids (biochanin A, eriocitrin, formononetin and hesperidin), indoles (indole and indole-3-carboxylic acid), mandelic acids (particularly 3-hydroxymandelic acid) and phenylpyruvic acid (phenylpyruvic acid) were found exclusively using water extraction. Conversely, certain as amines (spermidine), benzoic acids (2,3-dihydroxybenzoic acid, 2,4-dihydroxybenzoic acid, 2,5-dihydroxybenzoic acid, 2,6-dihydroxybenzoic acid, 3,4-dimethoxybenzoic acid and p-anisic acid), benzenes (particularly 1,2,3-trihydroxybenzene and 1,2-hydroxybenzene), coumarins (coumarin), flavonoids (bergapten, ethylferulate, imperatorin and niacin) and phenylpropionic acids (4-hydroxy-3-methoxyphenylpropionic acid and 4-hydroxyphenylpropionic acid) were exclusive to the organic extracted fractions (Fig. 1).

Common to both extraction procedures, catechin was found to be the most abundant phytochemical present at a concentration of 12248.7 ± 1594.3 ng/mg in the organic fraction, which was significantly (p < 0.05) lower in all six of the organic fractions than the aqueous fractions (cold and hot) at 40,220 ± 17790.8 ng/mg (Table 1). Also, significantly (p < 0.05) higher in the aqueous fractions were 3,4-dimethoxybenzaldehyde, 4-hydroxyphenylpyruvic acid, apigenin, benzoic acid, epigallocatechin gallate, gallocatechin, genstein, indole-3-carboxaldehyde, indole-3-pyruvic acid, kaempferol, luteolin, myricetin, naringenin, phloretin, phloridzin, p-hydroxybenzoic acid, protocatachaldehyde, protocatechuic acid, quercetin, quercetin-3-glucoside, resveratrol, scopoletin, syringaresinol and taxifolin. On the other hand, significantly (p < 0.05) lower amounts of anthranilic acid, phenyllactic acid and salicylic acid were found in the aqueous extracts (Table 1).

**Table 1:** Comparison of phytochemical profile from TI sample obtained in February 2018 and extracted using organic solvents or traditional (both cold and hot water) extraction methods

Phytochemical class	Phytochemical metabolites	Organic solvents extraction (ng/mg)	Organic solvents extraction relative abundance (%)	Traditional (aqueous) extraction (ng/mg)	Traditional (aqueous) extraction relative abundance (%)
Acetophenones	3,4,5-Trimethoxyacetophenone	2.2 ± 0.5	0.01	4.2 ± 1.9	0.01
	4-Hydroxy-3-methoxyacetophenone	12.4 ± 2.3	0.03	20.0 ± 4.5	0.03
	4-Hydroxyacetophenone	8.1 ± 3.7	0.02	44.2 ± 8.9**	0.06
Benzaldehydes	3,4-Dimethoxybenzaldehyde	3.3 ± 0.5	0.01	13.4 ± 4.7**	0.02
	P-Hydroxybenzaldehyde	20.6 ± 5.8	0.05	16.9 ± 5.4	0.02
	Protocatachaldehyde	68.8 ± 8.9	0.17	240.8 ± 52.2***	0.33
	Syringin	27.6 ± 4.3	0.07	25.8 ± 5.1	0.04
	Vanillin	135.8 ± 48.1	0.34	103.4 ± 23.8	0.14
Benzoic acids	Anthranilic acid	7.2 ± 0.9	0.02	3.6 ± 0.4*	0.00
	Benzoic acid	360.6 ± 173.8	0.90	793.8 ± 70.4**	1.09
	Chlorogenic acid	10.1 ± 2.5	0.03	11.2 ± 1.1	0.02
	Gallic acid	6199.3 ± 808.9	15.52	6520 ± 507.3	8.95
	P-hydroxybenzoic acid	34.2 ± 5.6	0.09	91 ± 25.3***	0.12
	Protocatechuic acid	266.9 ± 61.8	0.67	692 ± 237.5***	0.95
	Salicylic acid	6357.9 ± 1261.4	15.92	44.4 ± 17.0***	0.06
	Syringic acid	65.5 ± 13.4	0.16	55.3 ± 15.9	0.08
	Vanillic acid	145.6 ± 37.2	0.36	182 ± 43.9	0.25
Cinnamic acids	Caffeic acid	347.6 ± 62.2	0.87	211.8 ± 64.8	0.29
	Cinnamic acid	7.0 ± 2.1	0.02	6.6 ± 1.9	0.01
	Ferulic acid	255.9 ± 40.3	0.64	210.6 ± 51.7	0.29
	P-coumaric acid	151.6 ± 45.2	0.38	167.6 ± 34.6	0.23
	Sinapic acid	11.4 ± 4.8	0.03	7.6 ± 2.1	0.01
Flavonoids	Apigenin	12.8 ± 0.6	0.03	37.2 ± 16.9**	0.05
	Catechin	12248.7 ± 1594.3	30.67	40,220 ± 17790.8***	55.18
	Epicatechin	2806 ± 562.8	7.03	2264 ± 814.5	3.11
	Epigallocatechin	2152 ± 226.2	5.39	1968 ± 452.5	2.70
	Epigallocatechin gallate	581.2 ± 73.3	1.46	5240 ± 113.1***	7.19
	Gallocatechin	5340 ± 537.4	13.37	8360 ± 961.6**	11.47
	Genstein	15.3 ± 0.9	0.04	41.1 ± 17.6**	0.06
	Hesperitin	7.7 ± 2.2	0.02	8.4 ± 3.5	0.01
	Luteolin	14.8 ± 4.8	0.04	69.6 ± 14.4***	0.10
	Morin	25.1 ± 5.3	0.06	36.8 ± 5.8	0.05
	Myricetin	69.5 ± 16.6	0.17	198.2 ± 40.8***	0.27
	Naringenin	42.6 ± 14.8	0.11	232.4 ± 74.6***	0.32
	Neohesperidin	24.7 ± 5.3	0.06	29.6 ± 4.8	0.04
	Phloretin	42.4 ± 2.8	0.11	322.6 ± 137.7***	0.44
	Phloridzin	14.6 ± 5.1	0.04	116.4 ± 35.6***	0.16
	Psoralen	2.3 ± 0.5	0.01	1.6 ± 0.4	0.00
	Quercetin	63.7 ± 9.9	0.16	216.4 ± 65.6***	0.30
	Quercetin-3-glucoside	30 ± 5.8	0.08	133.2 ± 40.7***	0.18
	Reservatrol	17.9 ± 4.9	0.04	46.4 ± 6.7**	0.06
	Scopoletin	20.3 ± 4.4	0.05	78 ± 12.4***	0.11
	Tangeretin	10.5 ± 5.1	0.03	7.6 ± 4.4	0.01
	Taxifolin	73.5 ± 19.9	0.18	329.2 ± 145.3***	0.45
Indoles	Indole-3-acetic acid	21.0 ± 5.2	0.05	31.3 ± 5.4	0.04
	Indole-3-pyruvic acid	201.2 ± 51.7	0.50	1028 ± 400.1***	1.41
Lignans	Indole-3-carboxaldehyde	7.2 ± 2.6	0.02	51.6 ± 5.1***	0.07
	Pinoresinol	45.6 ± 10.7	0.11	35.1 ± 8.4	0.05
	Secoisolariciresinol	257.5 ± 65.1	0.64	199.4 ± 67.5	0.27
	Syringaresinol	540 ± 118.2	1.35	1114 ± 280***	1.53
Phenols	Hydroxytyrosol	90.6 ± 23.5	0.23	86.4 ± 31.1	0.12
Phenylacetic acids	Phenylacetic acids	16.2 ± 4.3	0.04	30.3 ± 5.8	0.04
Phenyllactic acids	Phenyllactic acid	20.3 ± 5.1	0.05	3.0 ± 1.8***	0.00
Phenylpyruvic acids	4-hydroxyphenylpyruvic acid	624 ± 69.6	1.56	882 ± 308.6**	1.21
	**SUM**	**39938.8**	**100**	**72884.0**	**100**

Data are presented as mean amount ± SD (ng/mg) and mean relative abundances (%), n = 3, *p < 0.05, **p < 0.01 and ***p < 0.001 as compared to organic solvents extraction of TI sample (obtained in dry season) by ANOVA followed by Bonferroni’s post hoc test

#### Effect of hot water versus cold water extraction on the phytochemical profile of TI

There was a total of 67 phytochemicals identified using both cold and hot water. There was a greater number of phytochemicals extracted using cold water compared to hot water extraction. Metabolites including cinnamic acids (3,4-dimethoxycinnamic acid and cinnamic acid), mandelic acids (3-hydroxymandelic acid), flavonoids (hesperidin, morin and neohesperidin), indoles (indole-3-carboxylic acid) and phenylpyruvic acids (phenylpyruvic acid) were present only in the cold-water extract, while amines (spermine), benzoic acids (anthranilic acid), flavonoids (eriocitrin and psoralen) or indoles (indole-3-pyruvic acid) were absent in the cold-water extract (Fig. 2).

Again, catechin was the most abundant phytochemical detected using both cold (52,800 ± 13,200 ng/mg) and hot water (27,640 ± 6910 ng/mg) extracts (Table 2).

**Table 2:** Comparison of phytochemical profile from TI sample obtained in February 2018 and extracted using cold versus hot water traditional extraction methods

Phytochemical class	Phytochemical metabolites	Hot water (ng/mg)	Hot water relative abundance (%)	Cold water (ng/mg)	Cold water relative abundance (%)
Acetophenones	3,4,5-Trimethoxyacetophenone	1.8 ± 0.4	0.00	6.5 ± 1.8***	0.01
	4-Hydroxy-3-methoxyacetophenone	6.8 ± 1.7	0.01	33.3 ± 8.2***	0.04
	4-Hydroxyacetophenone	8.0 ± 2.0	0.01	80.4 ± 20.1***	0.09
Amines	Serotonin	9.1 ± 2.2	0.02	5.0 ± 1.2	0.01
	Tyramine	0.7 ± 0.2	0.00	1.1 ± 0.4	0.00
Benzaldehydes	3,4-Dimethoxybenzaldehyde	10.0 ± 2.2	0.02	16.7 ± 4.1	0.02
	P-Hydroxybenzaldehyde	5.8 ± 1.4	0.01	28 ± 6.9***	0.03
	Protocatachaldehyde	133.2 ± 33.3	0.24	348.4 ± 87.1***	0.39
	Syringin	10.8 ± 2.4	0.02	40.8 ± 9.8***	0.05
	Vanillin	51.2 ± 12.8	0.09	155.6 ± 38.8***	0.18
Benzoic acids	Benzoic acid	107.6 ± 26.9	0.19	1480 ± 370***	1.67
	Chlorogenic acid	10.4 ± 2.6	0.02	11.9 ± 2.9	0.01
	Gallic acid	4040 ± 1010	7.30	9000 ± 2250***	10.15
	P-Hydroxybenzoic acid	44.8 ± 11.2	0.08	137.2 ± 34.3***	0.15
	Protocatechuic acid	524 ± 131	0.95	860 ± 215*	0.97
	Salicylic acid	32.3 ± 8.0	0.06	56.4 ± 14.1	0.06
	Syringic acid	36.9 ± 9.2	0.07	73.6 ± 18.4**	0.08
	Vanillic acid	109.2 ± 27.2	0.20	254.8 ± 63.7**	0.29
Cinnamic acids	4-Methoxycinnamic acid	7.8 ± 1.9	0.01	7.7 ± 1.9	0.01
	Caffeic acid	95.2 ± 23.8	0.17	328.4 ± 82.1***	0.37
	Ferulic acid	131.6 ± 32.9	0.24	289.6 ± 72.4***	0.33
	P-Coumaric acid	108.8 ± 27.2	0.20	226.4 ± 56.5**	0.26
	Sinapic acid	1.0 ± 0.2	0.00	14.2 ± 3.5***	0.02
Flavonoids	Apigenin	25.2 ± 6.3	0.05	49.2 ± 12.3*	0.06
	Biochanin A	16.3 ± 4.0	0.03	18.9 ± 4.7	0.02
	Catechin	27,640 ± 6910	49.95	52,800 ± 13,200**	59.52
	Epicatechin	2840 ± 710	5.13	1688 ± 422	1.90
	Epigallocatechin	2128 ± 532	3.85	1808 ± 452	2.04
	Epigallocatechin gallate	5320 ± 1330	9.61	5160 ± 1290	5.82
	Formononetin	3.1 ± 0.7	0.01	3.2 ± 0.8	0.00
	Gallocatechin	7680 ± 1920	13.88	9040 ± 2260	10.19
	Genstein	28.6 ± 7.1	0.05	53.6 ± 13.4*	0.06
	Hesperitin	5.9 ± 1.9	0.01	10.9 ± 2.7*	0.01
	Kaempferol	89.2 ± 22.3	0.16	184.8 ± 46.2**	0.21
	Luteolin	59.6 ± 14.9	0.11	79.6 ± 19.9	0.09
	Myricetin	210 ± 52.5	0.38	186.4 ± 46.6	0.21
	Naringenin	179.6 ± 44.9	0.32	285.2 ± 71.3*	0.32
	Phloretin	225.2 ± 56.3	0.41	420 ± 105*	0.47
	Phloridzin	91.2 ± 22.8	0.16	141.6 ± 35.4	0.16
	Quercetin	170 ± 42.5	0.31	262.8 ± 65.7*	0.30
	Quercetin-3-glucoside	83.6 ± 20.9	0.15	182.8 ± 45.7**	0.21
	Resveratrol	41.6 ± 10.4	0.08	51.2 ± 12.8	0.06
	Scopoletin	42 ± 10.5	0.08	114 ± 28.5***	0.13
	Tangeretin	10.7 ± 2.6	0.02	4.4 ± 1.1*	0.00
	Taxifolin	226.4 ± 56.6	0.41	432 ± 108**	0.49
Indoles	Indole	24.2 ± 16.1	0.04	110.4 ± 27.6***	0.12
	Indole-3-acetic acid	6.2 ± 1.5	0.01	56.4 ± 14.1***	0.06
Lignans	Indole-3-carboxaldehyde	19.6 ± 4.9	0.04	83.6 ± 20.9***	0.09
	Pinoresinol	35.0 ± 8.7	0.06	35.1 ± 8.7	0.04
	Secoisolariciresinol	151.6 ± 37.9	0.27	247.2 ± 61.8	0.28
	Syringaresinol	1312 ± 329	2.37	916 ± 229	1.03
Phenols	Hydroxytyrosol	64.4 ± 16.1	0.12	108.4 ± 27.1**	0.12
Phenylacetic acids	Phenylacetic acids	14.9 ± 3.7	0.03	45.6 ± 11.4***	0.05
Phenyllactic acids	Phenyllactic acid	1.7 ± 0.4	0.00	4.2 ± 1.0*	0.00
Phenylpyruvic acids	4-hydroxyphenylpyruvic acid	1100 ± 275	1.99	664 ± 166**	0.75
	**SUM**	**55331.8**	**88703.5**	**100**	**100**

Data are presented as mean amount ± SD (ng/mg) and mean relative abundances (%), n = 3, *p < 0.05, **p < 0.01 and ***p < 0.001 as compared to hot water extraction of TI sample (obtained in dry season) by ANOVA followed by Bonferroni’s post hoc test

Significantly (p < 0.05) higher concentrations of 3,4,5-trimethoxyacetophenone, 4-hydroxy-3-methoxyacetophenone, 4-hydroxyacetophenone, apigenin, benzoic acid, caffeic acid, catechin, ferulic acid, gallic acid, genstein, hydroxytyrosol, indole, indole-3-acetic acid, indole-3-carboxaldehyde, kaempferol, naringenin, p-coumaric acid, phenylacetic acid, phenyllacetic acid, phloretin, p-hydroxybenzaldehyde, p-hydroxybenzoic acid, protocatachaldehyde, protocatechuic acid, quercetin, quercetin-3-glucoside, scopoletin, sinapic acid, syringic acid, syringin, taxifolin, vanillic acid and vanillin were found in the cold water extract. In contrast, significantly (p < 0.05) higher concentrations of 4-hydroxyphenylpyruvic acid and tangeretin were found in the hot water extract (Table 2).

### Effect of season on the phytochemical profile of TI

There was a total number of 77 phytochemicals were identified across TI samples obtained in both the dry and rainy seasons. Certain metabolites were absent from the TI sample obtained in the rainy season. These included certain amines (spermidine), acetophenones (3,4,5-trimethoxyacetophenone), benzaldehydes (protocatachaldehyde), benzenes (1,2-dihydroxybenzene and 1,2,3-trihydroxybenzene), benzoic acids (2,3-dihydroxybenzoic acid, 2,4-dihydroxybenzoic acid, 2,5-dihydroxybenzoic acid, 2,6-dihydroxybenzoic acid, 3,4-dimethoxybenzoic acid and anthranilic acid), cinnamic acids (sinapic acid), coumarins (coumarin), flavonoids (ethylferulate, epigallocatechin, kaempferol, niacin and), indoles (indole-3-acetic acid), phenols (hydroxytyrosol), phenylacetic acids (phenylacetic acid), phenyllactic acids (phenyllactic acid) and phenylpropionic acids (4-hydroxyphenylpropionic acid). Only 2-hydroxybenzyl alcohol, biochanin A, didymin, dopamine, formononetin, hesperidin and indole were detected exclusively in the TI sample obtained in the rainy season (Table 3).

**Table 3:** Comparison of phytochemical profile from TI samples obtained in February 2018 (dry season) or September 2018 (rainy season) extracted using different organic solvents

Phytochemical class	Phytochemical metabolites	Petroleum ether extract (ng/mg)		Chloroform extract (ng/mg)		Ethyl acetate extract (ng/mg)		Ethanol extract (ng/mg)	
		Dry season	Rainy season	Dry season	Rainy season	Dry season	Rainy season	Dry season	Rainy season
Acetophenones	3,4,5-trimethoxyacetophenone	2.7 ± 0.6	ND	ND	ND	2.0 ± 0.2	ND	2.1 ± 0.5	ND
	4-hydroxy-3-methoxyacetophenone	36.4 ± 6.2	0.2 ± 0.1***	4.6 ± 1.1	ND	4.8 ± 1.1	ND	3.9 ± 0.9	ND
	4-hydroxyacetophenone	10.1 ± 2.1	ND	12.2 ± 2.8	4.1 ± 1.1**	5.3 ± 1.2	8.1 ± 1.8	4.6 ± 1.1	ND
Amines	Dopamine	ND	3.1 ± 0.7	ND	3.0 ± 0.7	ND	7.4 ± 1.8	ND	6.6 ± 1.6
	Spermidine	20.6 ± 5.1	ND	ND	ND	ND	ND	ND	ND
Benzaldehydes	3,4-dimethoxybenzaldehyde	4.9 ± 1.1	9.3 ± 1.3	1.7 ± 0.4	ND	ND	ND	ND	ND
	P-hydroxybenzaldehyde	ND	9.9 ± 2.4	34.4 ± 4.7	24.7 ± 5.1	ND	9.0 ± 2.2	6.6 ± 1.3	3.7 ± 0.9
	Protocatachaldehyde	ND	ND	54.8 ± 13.7	ND	36.7 ± 9.1	ND	114.8 ± 28.7	ND
	Syringin	21.2 ± 4.2	55.2 ± 6.5*	77.6 ± 14.2	57.2 ± 9.6	1.0 ± 0.1	17.2 ± 3.4***	10.4 ± 2.1	4.8 ± 1.2*
	Vanillin	132.8 ± 23.7	210.4 ± 23.8	359.6 ± 48.6	179.2 ± 23.3*	7.7 ± 1.4	48.2 ± 8.3*	43.2 ± 6.1	42.3 ± 5.6
Benzenes	1,2-dihydroxybenzene	ND	ND	ND	ND	984 ± 246	ND	ND	ND
	1,2,3-trihydroxybenzene	ND	ND	ND	ND	ND	ND	64.0 ± 16.0	ND
Benzoic acids	2,3-dihydroxybenzoic acid	14.2 ± 3.5	ND	ND	ND	58 ± 14.5	ND	ND	ND
	2,4-dihydroxybenzoic acid	18 ± 4.5	ND	ND	ND	ND	ND	ND	ND
	2,5-dihydroxybenzoic acid	ND	ND	ND	ND	22.1 ± 5.5	ND	ND	ND
	2,6-dihydroxybenzoic acid	ND	ND	ND	ND	5.2 ± 1.3	ND	ND	ND
	3,4-dimethoxybenzoic acid	ND	ND	ND	ND	99.6 ± 24.9	ND	ND	ND
	Anthranilic acid	ND	ND	ND	ND	6.8 ± 1.6	ND	7.6 ± 1.8	ND
	Benzoic acid	291.6 ± 29.3	165.6 ± 31.1*	620.1 ± 79.7	468.3 ± 72.2	281.6 ± 37.5	218.0 ± 34.6	249.2 ± 31.6	476 ± 49.8*
	Chlorogenic acid	ND	ND	ND	ND	ND	ND	10.1 ± 2.2	27.2 ± 3.4*
	Gallic acid	ND	ND	37.9 ± 9.4	ND	3200 ± 512.3	3880 ± 486.8	15,360 ± 4102	2684 ± 245.3***
	P-anisic acid	31.1 ± 4.8	26.3 ± 5.1	30.1 ± 4.6	18.6 ± 2.3	ND	27.2 ± 6.8	ND	18.4 ± 4.6
	P-hydroxybenzoic acid	ND	ND	55.6 ± 5.8	1.2 ± 0.4***	6.2 ± 1.8	22.1 ± 3.2	40.8 ± 3.6	7.8 ± 2.2*
	Protocatechuic acid	ND	ND	19.4 ± 4.8	ND	249.2 ± 25.6	134 ± 18.7*	532 ± 54.8	119.2 ± 32.4**
	Salicylic acid	25,280 ± 3026	71.2 ± 14.7***	39.1 ± 3.1	57.6 ± 8.2	76.4 ± 16.8	46.4 ± 13.3*	36.1 ± 4.8	50.8 ± 4.9
	Syringic acid	ND	0.1 ± 0.02	62.4 ± 12.1	37.6 ± 2.5	100.4 ± 21.5	17.1 ± 3.3***	33.7 ± 8.4	ND
	Vanillic acid	48.4 ± 6.9	39.8 ± 11.4	348.8 ± 47.8	96.4 ± 15.3**	84.8 ± 16.2	44.0 ± 8.1*	100.4 ± 17.3	29.2 ± 7.2***
Cinnamic acids	Caffeic acid	ND	ND	ND	ND	391.6 ± 42.8	23.5 ± 4.1***	303.6 ± 43.4	19.3 ± 4.2***
	Cinnamic acid	ND	3.3 ± 0.6	ND	5.0 ± 1.2	7.0 ± 1.7	ND	ND	ND
	Ferulic acid	15 ± 3.6	3.1 ± 0.7**	92.4 ± 8.3	54.4 ± 7.9	516.0 ± 52.7	52.4 ± 8.4***	400.9 ± 76.5	22.2 ± 3.2***
	P-coumaric acid	ND	ND	11.2 ± 2.2	11.4 ± 2.5	58.8 ± 6.8	54.8 ± 7.1	384.8 ± 34.8	38.8 ± 5.5***
	Sinapic acid	ND	ND	ND	ND	11.4 ± 2.8	ND	ND	ND
Coumarins	Coumarin	3.0 ± 0.6	ND	ND	ND	ND	ND	ND	ND
Flavonoids	Apigenin	ND	ND	12.7 ± 2.6	9.6 ± 2.9	12.2 ± 2.3	14.9 ± 3.4	13.4 ± 3.2	12.4 ± 3.4
	Bergapten	ND	ND	1.1 ± 0.2	0.8 ± 0.1	ND	1.7 ± 0.4	ND	ND
	Biochanin A	ND	14.3 ± 3.3	ND	ND	ND	ND	ND	ND
	Catechin	ND	ND	25.9 ± 3.4	32.5 ± 5.7	18,800 ± 4286.1	16,280 ± 3621.3	17,920 ± 3532	12,480 ± 2642.4
	Didymin	ND	9.4 ± 2.5	ND	9.2 ± 2.3	ND	10.1 ± 2.5	ND	10.2 ± 2.5
	Epicatechin	ND	ND	ND	ND	3204 ± 801	ND	2408 ± 147.7	56.2 ± 14.2***
	Epigallocatechin	ND	ND	ND	ND	2472 ± 618	ND	1832 ± 458	ND
	Epigallocatechin gallate	ND	ND	ND	ND	34.3 ± 3.9	165.2 ± 31.4***	1128 ± 122.4	114.4 ± 21.6***
	Ethylferulate	198.1 ± 49.6	ND	ND	ND	ND	ND	ND	ND
	Formononetin	ND	3.7 ± 0.9	ND	4.4 ± 1.1	ND	3.8 ± 0.9	ND	3.9 ± 0.9
	Gallocatechin	ND	ND	ND	ND	5720 ± 652.5	684 ± 55.8***	4960 ± 672.5	456 ± 59.8***
	Genstein	ND	ND	16.1 ± 2.1	13.2 ± 3.2	14.4 ± 4.1	16.9 ± 3.8	15.4 ± 2.8	15.0 ± 3.2
	Hesperidin	ND	ND	ND	ND	ND	24.2 ± 6.0	ND	22.8 ± 5.7
	Hesperitin	ND	17.4 ± 4.5	17.7 ± 3.5	82.6 ± 13.4***	3.3 ± 0.4	55.6 ± 5.1***	2.8 ± 0.3	57.6 ± 4.4***
	Imperatorin	ND	10.2 ± 2.5	3.5 ± 0.9	3.7 ± 0.7	ND	5.1 ± 1.2	5.6 ± 1.5	4.8 ± 1.4
	Kaempferol	ND	ND	27.1 ± 6.7	ND	35.0 ± 8.7	ND	56 ± 14	ND
	Luteolin	ND	ND	ND	ND	31.2 ± 2.5	33.2 ± 4.8	28.1 ± 5.2	31.8 ± 4.5
	Morin	ND	47.2 ± 11.2	100.4 ± 15.7	208.5 ± 34.2**	ND	82.0 ± 20.5	ND	82.8 ± 20.7
	Myricetin	ND	ND	ND	ND	138.8 ± 32.3	94.8 ± 19.8	139.2 ± 24.4	94.4 ± 21.2
	Naringenin	ND	ND	66.4 ± 11.2	77.2 ± 13.6	42.4 ± 6.7	114.8 ± 16.8***	61.6 ± 17.	99.2 ± 15.3**
	Neohesperidin	ND	ND	ND	10.6 ± 2.6	16.9 ± 2.5	25.9 ± 4.6	32.4 ± 5.7	124 ± 23.5***
	Niacin	ND	ND	ND	ND	322.8 ± 80.7	ND	54.8 ± 13.7	ND
	Phloretin	ND	ND	ND	3.8 ± 0.9	40.4 ± 5.2	64 ± 7.3	44.4 ± 5.3	34.1 ± 5.1
	Phloridzin	1.7 ± 0.4	ND	ND	ND	8.1 ± 1.2	75.2 ± 12.5***	34.0 ± 4.5	58.4 ± 6.2
	Psoralen	ND	ND	ND	ND	1.3 ± 0.3	ND	3.2 ± 0.7	2.6 ± 0.4
	Quercetin	ND	ND	31.9 ± 7.9	ND	68.8 ± 9.2	63.2 ± 8.4	90.4 ± 8.7	52.8 ± 6.3*
	Quercetin-3-glucoside	ND	ND	ND	ND	ND	65.6 ± 16.4	30.1 ± 3.8	51.2 ± 4.4
	Resveratrol	ND	ND	ND	ND	23.8 ± 5.4	5.1 ± 1.3*	12.7 ± 3.1	ND
	Tangeretin	6.5 ± 2.1	7.6 ± 2.2	12.8 ± 3.1	9.0 ± 2.7	5.9 ± 1.3	15.1 ± 2.4**	16.5 ± 3.4	13.1 ± 2.8
	Taxifolin	ND	ND	8.4 ± 2.1	ND	112 ± 25.6	135.6 ± 24.3	100 ± 19.7	111.2 ± 24.4
	Scopoletin	13.1 ± 3.3	1.5 ± 0.1***	6.3 ± 1.2	41.2 ± 4.4*	30.2 ± 7.5	ND	31.3 ± 7.8	ND
Indoles	Indole	ND	ND	ND	11.4 ± 2.8	ND	ND	ND	ND
	Indole-3-acetic acid	ND	ND	ND	ND	21.0 ± 5.2	ND	ND	ND
	Indole-3-pyruvic acid	ND	ND	ND	ND	201.2 ± 31.7	624 ± 121.7***	ND	844 ± 211
Lignans	Indole-3-carboxaldehyde	2.2 ± 0.2	2.6 ± 0.4	2.3 ± 0.4	9.6 ± 1.8*	7.0 ± 1.3	11.2 ± 2.4	17.2 ± 4.2	5.8 ± 1.3**
	Pinoresinol	ND	18.3 ± 4.5	45.6 ± 5.6	29.9 ± 3.7	ND	ND	ND	ND
	Secoisolariciresinol	ND	2.0 ± 0.5	468.2 ± 38.4	145.6 ± 23.3***	221.6 ± 39.7	112.8 ± 23.8*	82.8 ± 5.7	82.4 ± 8.2
	Syringaresinol	ND	ND	444.0 ± 50.2	440.0 ± 46.7	504 ± 126	ND	ND	ND
Phenols	2-hydroxybenzyl alcohol	ND	18.3 ± 4.5	ND	25.4 ± 6.2	ND	ND	ND	ND
	Hydroxytyrosol	ND	ND	ND	ND	159.2 ± 39.8	ND	ND	ND
Phenylacetic acids	Phenylacetic acids	12.2 ± 4.3	49.6 ± 6.5*	2.4 ± 0.7	34.4 ± 4.9**	37.3 ± 9.3	ND	12.9 ± 3.2	ND
Phenyllactic acids	Phenyllactic acid	ND	ND	ND	ND	40.4 ± 10.1	ND	0.9 ± 0.1	ND
Phenylpropionic acids	4-hydroxyphenylpropionic acid	ND	ND	368.0 ± 96.0	ND	ND	ND	ND	ND
	4-hydroxy-3-methoxyphenylpropionic acid	ND	1.8 ± 0.4	ND	17.5 ± 4.3	2.8 ± 0.3***	1.6 ± 0.3	ND	ND
Phenylpyruvic acids	4-hydroxyphenylpyruvic acid	540 ± 60.2	2796 ± 306.8***	708.2 ± 65.5	408.3 ± 40.7	5160 ± 1290	ND	ND	608 ± 152

Data are presented as amount ± SD, n = 3, *p < 0.05, **p < 0.01 and ***p < 0.001 as compared to TI sample obtained in the dry season by ANOVA followed by Bonferroni’s post hoc test. ND represent not detected (minimum detectable limit of 0.1 ng/mg)

Again, catechin was the most abundant phytochemical measured in TI samples obtained from both seasons (dry season 18,800 ± 4286.1 ng/mg, rainy season 16,280 ± 3621.3 ng/mg) although the differences were not significant. Significantly (p < 0.05) lower concentrations, of some phytochemicals, however, were found in the TI sample obtained in the rainy season as compared to TI obtained in the dry season. These included certain acetophenones (4-hydroxy-3-methoxyacetophenone), benzoic acids (gallic acid, p-hydroxybenzoic acid, protocatechuic acid, salicylic acid and vanillic acid), cinnamic acids (caffeic acid, ferulic acid and p-coumaric acid), flavonoids (epicatechin, gallocatechin, hesperitin, quercetin, resveratrol and scopoletin), indoles (indole-3-pyruvic acid), lignans (secoisolariciresinol) and phenylacetic acids (phenylacetic acid). Conversely, the TI sample obtained in the rainy season showed significantly (p < 0.05) higher concentrations of 4-hydroxy-3-methoxyphenylpropionic acid, benzoic acid, chlorogenic acid, morin, naringenin, phloridzin and syringin as compared to the sample obtained in the dry season (Table 3).

### Effect of storage on the phytochemical profile of TI

There was a total of 69 phytochemicals identified in both the fresh (September 2018) and stored (September 2014) TI samples obtained in the rainy season. Relative to the fresh TI sample, phytochemicals including 3,4-dihydroxyphenylpropionic acid, 3,4-dimethoxybenzoic acid, 4-hydroxyphenylpropionic acid, caffeine, eriocitrin, ethylferulate, indole-3-carboxylic acid, kaempferol, kynurenic acid, protocatachaldehyde, spermidine and spermine were found only in the stored TI sample. Moreover, storage of TI sample resulted in the loss of certain phytochemicals found in the fresh TI sample. These included certain benzoic acids (chlorogenic acid and p-anisic acid), cinnamic acid, flavonoids (biochanin A, didymin, epicatechin, formononetin, hesperidin, imperatorin and neohesperidin), 2-hydroxybenzyl alcohol and phenylacetic acid which were lost due to storage of the TI sample (Table 4).

**Table 4:** Comparison of phytochemical profile from TI samples obtained in September 2014 (stored) or September 2018 (fresh) extracted using different organic solvents

Phytochemical class	Phytochemical metabolites	Petroleum ether extract (ng/mg)		Chloroform extract (ng/mg)		Ethyl acetate extract (ng/mg)		Ethanol extract (ng/mg)	
		Fresh sample	Stored sample	Fresh sample	Stored sample	Fresh sample	Stored sample	Fresh sample	Stored sample
Acetophenones	4-hydroxy-3-methoxyacetophenone	0.2 ± 0.1	ND	ND	43.2 ± 10.8	ND	ND	ND	ND
	4-hydroxyacetophenone	ND	ND	4.1 ± 1.1	64.0 ± 16.0***	8.1 ± 1.8	36.9 ± 5.1*	ND	ND
Amines	Dopamine	3.1 ± 0.7	3.7 ± 0.9	3.0 ± 0.7	3.7 ± 0.9	7.4 ± 1.8	ND	6.6 ± 1.6	ND
	Spermidine	ND	190.4 ± 47.6	ND	ND	ND	ND	ND	ND
	Spermine	ND	16.8 ± 4.1	ND	8.2 ± 2.0	ND	ND	ND	ND
Benzaldehydes	3,4-dimethoxybenzaldehyde	9.3 ± 1.3	ND	ND	4.3 ± 1.1	ND	8.2 ± 2.0	ND	10.5 ± 2.6
	P-hydroxybenzaldehyde	9.9 ± 2.4	ND	24.7 ± 5.1	121.6 ± 30.4***	9.0 ± 2.2	37.4 ± 8.1*	3.7 ± 0.9	8.0 ± 2.0*
	Protocatachaldehyde	ND	ND	ND	284.8 ± 71.2	ND	278.8 ± 69.7	ND	96.8 ± 24.2
	Syringin	55.2 ± 6.5	9.1 ± 2.2***	57.2 ± 9.6	271.2 ± 52.8***	17.2 ± 3.4	110.1 ± 16.8***	4.8 ± 1.2	32.1 ± 8.0***
	Vanillin	210.4 ± 23.8	147.6 ± 36.9	179.2 ± 23.3	1276 ± 319***	48.2 ± 8.3	416 ± 42.7***	42.3 ± 5.6	138 ± 34.5***
Benzoic acids	3,4-dimethoxybenzoic acid	ND	ND	ND	30 ± 7.5	ND	ND	ND	ND
	Benzoic acid	165.6 ± 31.1	130.4 ± 32.6	468.3 ± 72.2	1204 ± 301***	218.0 ± 34.6	74.8 ± 9.9*	476 ± 49.8	209.2 ± 52.3***
	Chlorogenic acid	ND	ND	ND	ND	ND	ND	27.2 ± 3.4	ND
	Gallic acid	ND	ND	ND	78 ± 19.5	3880 ± 486.8	14,720 ± 1525.6*	2684 ± 245.3	6080 ± 1520***
	P-anisic acid	26.3 ± 5.1	ND	18.6 ± 2.3	ND	27.2 ± 6.8	ND	18.4 ± 4.6	ND
	P-hydroxybenzoic acid	ND	ND	1.2 ± 0.4	149.6 ± 37.4***	22.1 ± 3.2	157.6 ± 16.9***	7.8 ± 2.2	40.8 ± 9.6***
	Protocatechuic acid	ND	ND	ND	162 ± 40.5	134 ± 18.7	1900 ± 354.6***	119.2 ± 32.4	656 ± 164***
	Salicylic acid	71.2 ± 14.7	ND	57.6 ± 8.2	142.8 ± 35.7***	46.4 ± 13.3	54.4 ± 8.3	50.8 ± 4.9	21.6 ± 5.4**
	Syringic acid	0.1 ± 0.02	ND	37.6 ± 2.5	234 ± 58.5***	17.1 ± 3.3	110.4 ± 23.5***	ND	31.6 ± 7.8
	Vanillic acid	39.8 ± 11.4	33.6 ± 8.4	96.4 ± 15.3	1200 ± 300***	44.0 ± 8.1	428 ± 40.1***	29.2 ± 7.2	134.4 ± 33.6***
Cinnamic acids	Caffeic acid	ND	ND	ND	ND	23.5 ± 4.1	42.8 ± 8.7	19.3 ± 4.2	8.4 ± 2.1*
	Cinnamic acid	3.3 ± 0.6	ND	5.0 ± 1.2	ND	ND	ND	ND	ND
	Ferulic acid	3.1 ± 0.7	ND	54.4 ± 7.9	400 ± 96.4***	52.4 ± 8.4	135.2 ± 15.9***	22.2 ± 3.2	32.8 ± 8.2
	P-coumaric acid	ND	ND	11.4 ± 2.5	97.6 ± 21.7***	54.8 ± 7.1	143.2 ± 21.3***	38.8 ± 5.5	48.8 ± 12.2
Flavonoids	Apigenin	ND	ND	9.6 ± 2.9	14.8 ± 3.7	14.9 ± 3.4	15.4 ± 3.1	12.4 ± 3.4	15.2 ± 3.8
	Bergapten	ND	0.8 ± 0.2	0.8 ± 0.1	0.8 ± 0.2	1.7 ± 0.4	ND	ND	ND
	Biochanin A	14.3 ± 3.3	ND	ND	ND	ND	ND	ND	ND
	Catechin	ND	115.6 ± 28.9	32.5 ± 5.7	37.7 ± 9.4	16,280 ± 3621.3	14,000 ± 1261.2	12,480 ± 2642.4	5640 ± 1410***
	Didymin	9.4 ± 2.5	ND	9.2 ± 2.3	ND	10.1 ± 2.5	ND	10.2 ± 2.5	ND
	Epicatechin	ND	ND	ND	ND	ND	ND	56.2 ± 14.2	ND
	Epigallocatechin gallate	ND	ND	ND	ND	165.2 ± 31.4	86.8 ± 16.4*	114.4 ± 21.6	42 ± 10.5***
	Eriocitrin	ND	ND	ND	ND	ND	5.8 ± 1.4	ND	ND
	Ethylferulate	ND	23.4 ± 5.8	ND	8.3 ± 2.0	ND	ND	ND	ND
	Formononetin	3.7 ± 0.9	ND	4.4 ± 1.1	ND	3.8 ± 0.9	ND	3.9 ± 0.9	ND
	Gallocatechin	ND	29.2 ± 7.2	ND	ND	684 ± 55.8	800 ± 67.9	456 ± 59.8	273.2 ± 68.3**
	Genstein	ND	ND	13.2 ± 3.2	18 ± 4.5	16.9 ± 3.8	20.9 ± 3.6	15.0 ± 3.2	17.0 ± 4.2
	Hesperidin	ND	ND	ND	ND	24.2 ± 6.0	ND	22.8 ± 5.7	ND
	Hesperitin	17.4 ± 4.5	ND	82.6 ± 13.4	21.9 ± 5.4***	55.6 ± 5.1	15.4 ± 3.3***	57.6 ± 4.4	8.2 ± 2.0***
	Imperatorin	10.2 ± 2.5	ND	3.7 ± 0.7	ND	5.1 ± 1.2	ND	4.8 ± 1.4	ND
	Kaempferol	ND	ND	ND	35.4 ± 8.8	ND	36.9 ± 9.2	ND	44.4 ± 11.1
	Kynurenic acid	ND	ND	ND	ND	ND	ND	ND	36.1 ± 9.0
	Luteolin	ND	ND	ND	ND	33.2 ± 4.8	59.2 ± 8.3	31.8 ± 4.5	50 ± 12.5
	Morin	47.2 ± 11.2	ND	208.5 ± 34.2	98 ± 19.5*	82.0 ± 20.5	51.2 ± 7.1*	82.8 ± 20.7	ND
	Myricetin	ND	99.2 ± 24.8	ND	96.8 ± 24.2	94.8 ± 19.8	104.4 ± 8.6	94.4 ± 21.2	101.2 ± 25.3
	Naringenin	ND	ND	77.2 ± 13.6	106 ± 22.8	114.8 ± 16.8	122.4 ± 10.3	99.2 ± 15.3	105.6 ± 26.4
	Neohesperidin	ND	ND	10.6 ± 2.6	ND	25.9 ± 4.6	ND	124 ± 23.5	ND
	Phloretin	ND	ND	3.8 ± 0.9	15.0 ± 3.5**	64 ± 7.3	65.6 ± 5.5	34.1 ± 5.1	26.2 ± 6.5
	Phloridzin	ND	ND	ND	ND	75.2 ± 12.5	158.8 ± 24.2*	58.4 ± 6.2	19.7 ± 4.9***
	Psoralen	ND	ND	ND	ND	ND	ND	2.6 ± 0.4	3.6 ± 0.9
	Quercetin	ND	34.6 ± 8.6	ND	48.8 ± 12.2	63.2 ± 8.4	125.2 ± 16.7**	52.8 ± 6.3	90.4 ± 22.6**
	Quercetin-3-glucoside	ND	ND	ND	ND	65.6 ± 16.4	94.2 ± 12.6*	51.2 ± 4.4	25.8 ± 6.4*
	Resveratrol	ND	ND	ND	ND	5.1 ± 1.3	8.4 ± 1.4	ND	13.3 ± 3.3
	Tangeretin	7.6 ± 2.2	8.0 ± 2.0	9.0 ± 2.7	9.4 ± 2.3	15.1 ± 2.4	6.9 ± 1.2	13.1 ± 2.8	10.1 ± 2.5
	Taxifolin	ND	ND	ND	31.7 ± 7.9	135.6 ± 24.3	211.2 ± 28.4*	111.2 ± 24.4	207.2 ± 51.8**
	Scopoletin	1.5 ± 0.1	ND	41.2 ± 4.4	2.7 ± 0.6***	ND	ND	ND	ND
Indoles	Indole	ND	ND	11.4 ± 2.8	6.2 ± 1.5*	ND	ND	ND	ND
	Indole-3-carboxylic acid	ND	ND	ND	3.1 ± 0.7	ND	ND	ND	ND
	Indole-3-pyruvic acid	ND	ND	ND	ND	624 ± 121.7	724 ± 43.8	844 ± 211	1400 ± 350*
Lignans	Indole-3-carboxaldehyde	2.6 ± 0.4	ND	9.6 ± 1.8	5.2 ± 1.3	11.2 ± 2.4	2.6 ± 0.3**	5.8 ± 1.3	6.0 ± 1.5
	Pinoresinol	18.3 ± 4.5	ND	29.9 ± 3.7	60.4 ± 15.1**	ND	23.7 ± 5.9	ND	62.4 ± 15.6
	Secoisolariciresinol	2.0 ± 0.5	ND	145.6 ± 23.3	572 ± 143***	112.8 ± 23.8	261.2 ± 34.5**	82.4 ± 8.2	249.6 ± 62.4***
	Syringaresinol	ND	ND	440.0 ± 46.7	496 ± 124	ND	ND	ND	524 ± 131
Phenols	2-hydroxybenzyl alcohol	18.3 ± 4.5	ND	25.4 ± 6.2	ND	ND	ND	ND	ND
	Caffeine	ND	ND	ND	3.9 ± 0.9	ND	ND	ND	ND
Phenylacetic acids	Phenylacetic acid	49.6 ± 6.5	ND	34.4 ± 4.9	ND	ND	ND	ND	ND
Phenylpropionic acids	3,4-dihydroxyphenylpropionic acid	ND	ND	ND	66.4 ± 16.6	ND	ND	ND	ND
	4-hydroxyphenylpropionic acid	ND	ND	ND	1388 ± 347	ND	664 ± 166	ND	ND
	4-hydroxy-3-methoxyphenylpropionic acid	1.8 ± 0.4	ND	17.5 ± 4.3	14.6 ± 3.6	1.6 ± 0.3	ND	ND	ND
Phenylpyruvic acids	4-hydroxyphenylpyruvic acid	2796 ± 306.8	664 ± 166***	408.3 ± 40.7	504 ± 126	5160 ± 1290	ND	608 ± 152	1560 ± 390***

Data are presented as amount ± SD, n = 3, *p < 0.05, **p < 0.01 and ***p < 0.001 as compared to fresh TI sample by ANOVA followed by Bonferroni’s post hoc test. ND represent not detected (minimum detectable limit of 0.1 ng/mg)

Again, catechin was the most abundant phytochemical measured in fresh TI sample (16,280 ± 3621.3 ng/mg) and gallic acid was the most abundant phytochemical present in stored TI sample (14,720 ± 1525.6 ng/mg) although the differences were not significant.

The stored TI sample showed significantly (p < 0.05) higher concentrations of acetophenones (4-hydroxyacetophenone, benzaldehydes p-hydroxybenzaldehyde), benzoic acids (gallic acid, p-hydroxybenzoic acid, protocatechuic acid and syringic acid), cinnamic acids (ferulic acid and p-coumaric acid), flavonoids (phloretin and tangeretin) and indoles (indole-3-pyruvic acid). Conversely, the stored TI sample showed significantly (p < 0.05) lower concentrations of certain flavonoids (catechin, epigallocatechin gallate, gallocatechin, hesperitin, morin, scopoletin, and taxifolin), indole and indole-3-carboxaldehyde when compared with the fresh sample (Table 4).

### Principal components analysis of TI samples

Principal component analysis (PCA) on a univariate scale was used to assess differences in the main phytochemical metabolites extracted from the TI samples (obtained in September 2014, February 2018 and September 2018 which were represented as 1, 2 and 3 respectively). Similarity in phytochemicals between samples was shown by how closely clustered they were to each other in the same quadrant of a plot. From the PCA plot (Supplementary Fig. 1A), sample obtained in February 2018 (2) was different from those obtained in September 2014 and September 2018 when extracted using water (hot and cold), ethanol or ethyl acetate. Looking across the 3 samples, similar metabolites were obtained from petroleum ether extraction. Metabolites from samples obtained in September 2014 and September 2018 were observed to be more similar by using all the solvents: water (hot or cold), ethanol, ethyl acetate except chloroform.

## DISCUSSION

Plants are essentially immobile in nature and cannot escape unfavourable environmental conditions. However, they produce secondary metabolites through various physiological and biochemical processes that improve their chances of survival and growth in response to changes in the environment [41, 42]. The type and concentration of secondary metabolites produced by a plant are species-dependent, are influenced by developmental stage, and by environmental conditions during growth [43]. The physiological processes involved in the synthesis of phytochemicals result in alterations in gene expression, regulation of protein activity, ion homeostasis and endogenous levels of phytochemicals [42]. For centuries, humans have exploited such physiological changes in plants for drug discovery or by directly using the plants as medicines or using metabolites from the plants to produce synthetic drugs [44]. TI is a tree that grows in the tropical and sub-tropical regions of the world and is mostly used for medicinal purposes [43]. This study focused on the impact of solvent (aqueous versus organic solvent) extraction on the phytochemical profile of TI and also investigated the influence of change in season (dry and rainy) and storage on secondary metabolites for its implication on traditional medicine.

Successful isolation of biologically active compounds from a plant material is principally dependent on the type of solvent used in the extraction procedure [45]. In scientific research, samples are frequently extracted with organic solvents like acetone, chloroform, dichloromethane, ethyl acetate, ethanol, petroleum ether [46–48]. Different solvent extracts of plant samples contain different phytochemicals and hence, have different biological activity [46, 47]. For example, water and ethanol extracts of several plants including *Hypoxia hemerocallidea, Ocimum basilicum* and *Senna petersiana* have higher antibacterial and antioxidant activity than other extracts such as chloroform or dichloromethane [47, 49] Traditional medicines are not generally extracted with chemical solvents but are rather routinely extracted using available solvents such as water or alcohol, and this results in differences in biological activity between extracts from traditional and chemical methods [50]. Therefore, this study compared phytochemicals isolated using traditional method of aqueous extraction (hot and cold water) with organic sequential solvent extraction.

The organic solvent extraction produced a wider profile of phytochemicals as compared to aqueous extraction, with 20% and 14% of the total phytochemicals being identified exclusively in the organic or aqueous extracts respectively. However, for the phytochemicals common to both extraction methods, water extraction showed an approximately 44% higher concentration of phytochemicals than the organic solvent extraction method (Table 1). Assuming the bioactive constituents are present using the traditional extraction methods, then no further benefits are likely to be realised by utilising solvent extraction.

Selection of a good solvent for extraction should be based on a high yield of target compounds, but should also allow for ease of subsequent handling of the extracts and minimal deterioration or metabolism of the compounds present [45]. Traditional extraction with cold and hot water showed higher amounts of several phytochemicals, while the sequential organic extraction with chloroform, ethanol, ethyl acetate and petroleum ether showed a wider profile of phytochemicals extracted. This could suggest metabolism of parent compounds during the extraction procedure, again advocating benefits of the traditional extraction methods. In addition to polarity of the extraction solvent, composition and yield of phytochemicals are highly dependent on temperature and duration of extraction [51, 52]. For this reason, the two most commonly used traditional extraction methods: hot and cold-water extraction. There was a greater number of secondary metabolites detected following cold-water extraction with significantly (p < 0.05) higher amounts of certain acetophenones, benzaldehydes, benzoic acids, cinnamic acids, flavonoids, indoles, lignans and and only a few compounds (4-hydroxyphenylpyruvic acid and tangeretin) being significantly (p < 0.05) lower in the cold-water extract. The decrease in number of secondary metabolites detected with hot water extraction, as for sequential Soxhlet extraction again could be due to the loss or metabolism of thermo-unstable compounds.

Analysis of the influence of season on TI secondary metabolites showed that 82 phytochemical metabolites were commonly identified from TI samples obtained in both the dry and the rainy seasons. Four organic solvents were used to obtain different phytochemical profiles from the TI samples. A total number of 85 and 69% of the phytochemicals were isolated from the TI sample obtained in the dry and rainy seasons respectively. As many as 24% of the 82 phytochemicals were found exclusively in the sample obtained in the dry season, while only 8% were identified exclusively in TI sampled in the rainy season.

During the dry season, there is a decrease in water and nutrient supply to plants [53]. Nutritional stress can result in the accumulation of osmo-protectants to stabilise proteins structure and maintain membrane integrity and scavenge reactive oxygen species (ROS), with biomass and secondary metabolites production [53, 54]. Phenolic compounds are involved in plant reproduction, growth and tolerance of stress [55, 56]. Moreover, plants that produce phenolics with allelopathic activity can compete with and suppress the growth of surrounding plants [57]. Phenolics also play other essential functions in plants such as indicators of stress, nutrient uptake, photosynthesis, and protein synthesis [58, 59]. There are a wide variety of compounds classified as phenolics which include coumarins, flavonoids, cinnamic acids and lignans [60].

Plant hormones such as auxins, salicylic acid, cytokinin, ethylene, gibberellic acid and jasmonic acid act to modulate developmental processes in plants and determine plant responses to environmental stresses [42, 59, 61]. In agreement with previous findings, this study observed higher levels of salicylic acid in TI collected in the more stressful dry season when compared to that obtained in the rainy season. The characteristic water deficit of the dry season has also been associated with induction of the synthesis of flavonoids, anthocyanins and phenolic acids in fruits and vegetables such as grape, lettuce, pomegranate and red beet [37]. This work also found that certain amines, acetophenones, benzaldehydes, benzenes, benzoic acids, cinnamic acids, coumarins, flavonoids, indoles, phenols, phenylacetic acids, phenyllactic acids and phenylpropionic acids exclusively in the TI sample obtained in dry season. Absence of adequate amounts of water or higher transpiration rates also result in drought stress in plants and changes secondary metabolite production [42, 61]. In contrast, a deficit in irrigation has been observed to reduce total anthocyanins and total phenolics in pomegranate [62].

Conversely, the rainy season is characterised by cloudy weather and relatively lower temperatures as compared to the sunny and high temperatures (up to 35–40 °C) observed in the dry season [23]. In this study, significantly (p < 0.05) higher amounts of cinnamic acids (chlorogenic acid), among other compounds were measured in TI sampled in the rainy season showed as compared to dry season.

In agreement with this study, a previous study reported that cooler weather was linked with the production of high levels of chlorogenic acid [63]. Plants growing in lower temperatures develop significant adjustments in several physiological and biochemical processes that enable them to survive under low temperature stress, and this causes inhibition in the synthesis and storage of secondary metabolites [64]. Moreover, water uptake, dehydration and metabolism in plants are reduced at low temperatures [65]. The light intensity and exposure period also have significant influence on the production and storage of secondary metabolites [66]. Coumarin levels have been shown to significantly decrease in different plant parts due to shorter light period [67].

Medicinal plant gatherers or traders usually wait to collect sufficient plant stock before supplying the market [68]. In the chain of production of medicinal products, herbalists usually store medicinal plant samples for days up to several months before use [35, 38]. Many of the bioactive compounds from these medicinal products may degrade during storage and where possible, it is recommended that plant samples should be extracted and analysed shortly after collection, as secondary metabolites can decompose even when stored under liquid nitrogen [69]. Analysing fresh and stored (4 years) TI samples, a total of 69 metabolites were identified common to both obtained in the rainy season. Further 12 different phytochemicals were detected exclusively in the fresh TI sample (Table 4) suggesting that these may be degraded during storage.

To the best of our knowledge, there are no data investigating metabolite profiles in fresh and stored TI, and hence, published findings describing the effect of storage on phytochemicals come from other plant sources. Storage of *Lilium* bulbs for 30 days results in a decrease in free amino acids, total polysaccharides and reducing sugars by approximately 39, 63 and 18% respectively [70]. It has also been reported that storage of *Cosmos caudatus* at room temperature for 12 h causes depletion of phenolic compounds (such as α-tocopherol, benzoic acid, catechin, cyclohexen-1-carboxylic acid, lycopene, myoinositol and stigmasterol) with phenolic compounds degraded into free sugars such as α-D-galactopyranose, sucrose and turanose, [36]. The depletion of phenolic compounds on storage is attributed to plant dehydration which occurs postharvest [71]. In the presence of oxygen, polyphenol oxidase converts phenolic compounds to quinones [72]. The activity of polyphenol oxidase in *Lilium* bulbs has been shown to increase by approximately 100% after 30 ays of storage [70]. Traditional healers commonly prefer fresh plant material because of doubts around the degree of biological activity of stored plants [49].

Contrary to the negative considerations about storage of plant material, modification of secondary metabolites during storage is not always detrimental and the increased activity or levels of specific compounds may be associated with a higher value product [47]. This study observed that higher levels of gallic acid (a natural antioxidant), and caffeine (a central nervous system stimulant) were present in the stored TI sample. Monribot-Villanueva et al. (2019) also found that gallic acid levels were elevated in some varieties of mango fruits after 6 days of storage [73].

Many traditional medical practitioners consider that the efficacy of stored plant material does not vary [50]. The results obtained from this current study, and other published findings confirms this since this study showed that 84% of the metabolites are still present after 4 years of storing TI bark sample.

Plant parts such as bark, roots or underground storage organs (such as bulbs, corms, rhizomes and tubers) may have a longer storage life compared to fruits or leaves while the compounds present within are more stable [49]. Moreover, these organs are better suited to protect the stored phytochemicals from degradation due to their lower surface area to volume ratio [47]. This confirms the findings of this study where TI bark that was stored for 4 years retained most of its secondary metabolites. However, while stored TI bark retained approximately 84% of econdary metabolites, fresh TI sample contained higher amounts. It was also found that the two TI samples obtained in the rainy season were more similar to each other but different from the TI sample obtained in the dry season.

### Conclusion

Method of extraction including temperature and solvent selection impacted on both the profile and quantity of metabolites measured in the extracts. In general, the cold water extracted sample had the highest amount of phytochemicals, albeit it with a narrower profile (compare to organic extracted) indicating different solubilities and thermo-stability of the metabolites. Higher phytochemical levels were also measured in the TI sample collected in the dry rather than wet season, likely due to harsher environmental conditions which is known to induce the synthesis and storage of secondary protective metabolites. TI that was stored for 4 years retained the majority of secondary metabolites measured in freshly collected TI, which could suggest a longer shelf-life for TI. This may be due to the ability of stem bark to provide a greater protection of the secondary metabolites thereby offering the metabolites more stability against the environment and hence, offering the medicinal product more stability. However, until the bioactive molecule (or molecules) responsible for the desired therapeutic effect are identified, optimal conditions for preparation cannot be fully demonstrated. This work, however, provides important information on composition and how this is modified by growing conditions, storage and method of extraction informing progress on the development of TI as a prophylactic formulation or medicine.

## Electronic supplementary material

Below is the link to the electronic supplementary material.

Supplementary Material 1

## Data Availability

The data can be made available from the corresponding author on reasonable request.
