# Supplementary material for: Season, storage and extraction method impact on the phytochemical profile of Terminalia ivorensis
Source: BMC Plant Biol. 2023 Mar 25;23:162. doi: 10.1186/s12870-023-04144-8 (PMC10039578; doi:10.1186/s12870-023-04144-8)
Supplement: Supplementary file 1 — Supplementary Material 1 [file 12870_2023_4144_MOESM1_ESM.docx]

**Supplementary Information 1A: Principal Component Analysis of TI samples**


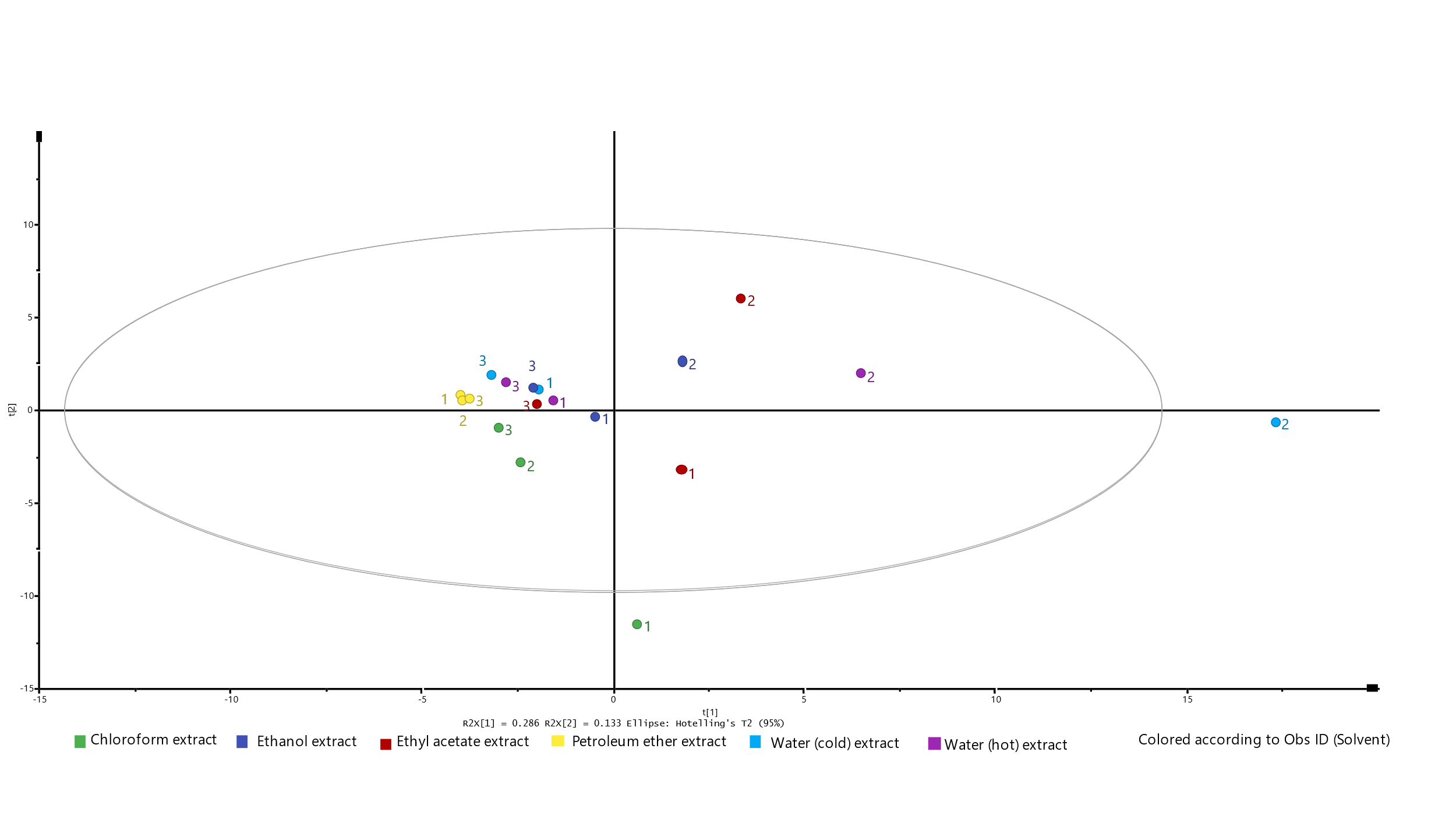
Figure 1A: Principal components analysis of TI samples obtained in September 2014 (represented as 1), February 2018 (represented as 2) and September 2018 (represented as 3) which were extracted with different organic solvents or water (hot or cold).
